# Nonphotochemical quenching in plants: Mechanisms and mysteries

**DOI:** 10.1093/plcell/koaf240

**Published:** 2025-10-07

**Authors:** Herbert van Amerongen, Roberta Croce

**Affiliations:** Laboratory of Biophysics, Wageningen University, Wageningen 6708 WE, the Netherlands; Biophysics of Photosynthesis, Department of Physics and Astronomy, Faculty of Science, Vrije Universiteit Amsterdam, Amsterdam 1081 HZ, the Netherlands

## Abstract

Plants are vulnerable to photodamage when exposed to light intensities that exceed their photosynthetic capacity. To protect themselves, they activate nonphotochemical quenching (NPQ), a set of processes that dissipate excess excitation energy as heat. NPQ has been studied extensively; however, the field remains conceptually fragmented, and consensus on the underlying mechanisms has yet to be reached. Interest in NPQ has recently intensified due to studies showing that tuning NPQ regulation can lead to substantial improvements in photosynthetic efficiency and even crop yield increases of up to 30%. In this review, we aim to bring structure to the diverse and sometimes contradictory NPQ literature by framing the discussion around a set of key mechanistic questions. We focus on the fastest component of NPQ, known as qE, which is activated within the first minutes of excess light exposure. Topics addressed include the molecular properties and roles of PsbS and zeaxanthin, potential conformational changes in light-harvesting complexes, reorganization of the thylakoid membrane, and the interplay among these factors. We synthesize the available evidence into a working model in which qE arises largely from a localized conformational switch in a small number of antenna complexes, triggered by PsbS, whereas zeaxanthin increases the domain size of the antenna that can be quenched by each of these quenchers.

## Photosynthesis and nonphotochemical quenching in a nutshell

**Figure 1.**
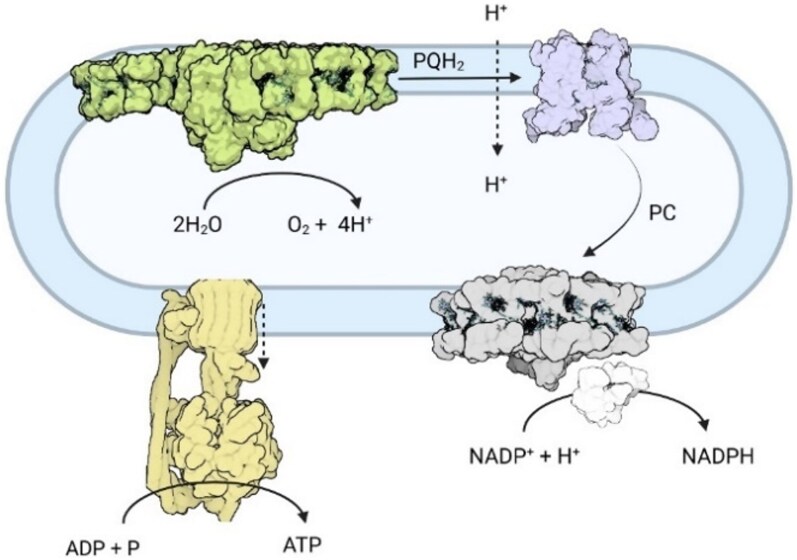
Cartoon of the thylakoid membrane, harboring the 4 major players in the light reactions: PSII (green), cyt b_6_f (purple), PSI (gray), and ATP synthase (yellow). This schematic drawing of a thylakoid membrane in the chloroplast of a plant cell shows the separation between the outside of the thylakoid membrane (stroma) and its inside (lumen). Light absorption in PSII and PSI leads to charge separation in their reaction centers, which enable the linear electron chain to extract electrons from H_2_O and eventually reduce NADP^+^ to form NADPH. The splitting of H_2_O at PSII increases the proton accumulation in the lumen, whereas the electron transport chain additionally contributes to pumping protons from the stroma (at the level of PSII and cyt b_6_f) to the lumen (at the level of cyt b_6_f). The ATP synthase uses the proton gradient to produce ATP from ADP and inorganic phosphate (P). Together ATP and NADPH fuel the dark reactions—in particular, the Calvin-Benson-Bassham cycle. The proton gradient created by light absorption can induce NPQ.

Oxygenic photosynthesis is the biological process by which green plants, algae, and certain bacteria convert light energy into chemical energy, storing it in the bonds of carbohydrates. At the heart of this process are the light reactions (Fig. 1), which occur in the thylakoid membranes and produce ATP and NADPH, the essential energy carriers for the subsequent synthesis of organic molecules during the Calvin-Benson-Bassham cycle (for a comprehensive description of the process, see Blankenship 2021).

**Figure 2.**
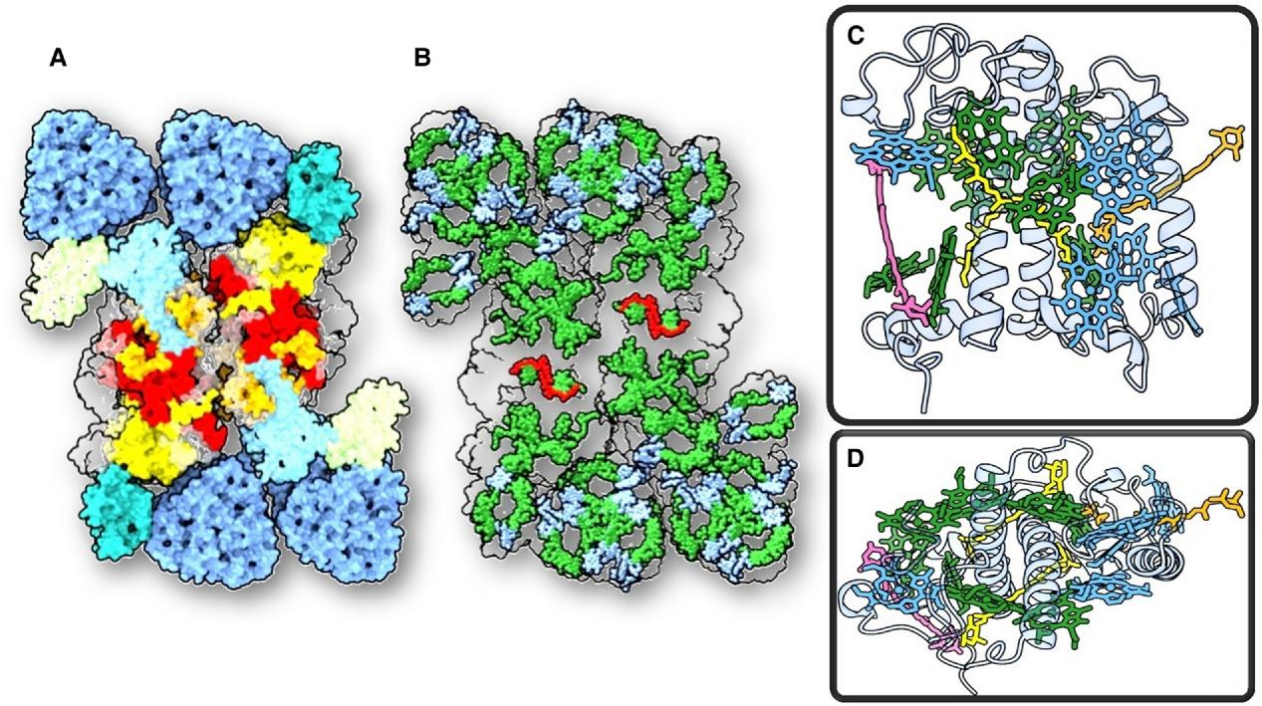
Structure of dimeric PSII supercomplex and monomeric LHCII. **A)** Structure of a dimeric PSII supercomplex from pea containing 4 LHCII trimers (blue) and 2× 3 minor light-harvesting complexes—CP26 (turquoise), CP29 (light blue), and CP24 (light green)—together constituting the outer antenna (Su et al. 2017). The 2 core complexes consist of the D1/D2 reaction center proteins and their pigments (red) and the core antenna proteins CP43 and CP47 (yellow and orange, respectively); all other PSII components are gray. The structure shows a cleft between the core proteins and CP24, which has been suggested as a place where PsbS might bind during NPQ. **B)** Structure of the same PSII supercomplex as in panel A but with the Chls *a* (green) and Chls *b* (blue) being highlighted. The Chls *a* in both PSII RCs are red. The structures in panels A and B are views from the stromal side of the membrane. **C)** Cartoon representation of a monomeric LHCII subunit of an LHCII trimer from spinach (Liu et al. 2004) viewed from within the plane of the membrane. Chl *a* molecules are green and Chls *b* in blue. The magenta Car is Vx, neoxanthin (Nx) is orange, and the central luteins are yellow. The lutein that is more or less parallel to Nx is L1. **D)** The same LHCII monomer is shown as seen from the stromal side.

The light reactions are mediated by 2 types of photosystems—photosystem I (PSI) and photosystem II (PSII)—each containing a reaction center (RC), where light energy is used to drive charge separation, and an antenna system that increases light absorption. There are two types of antenna systems: the inner antenna, which is directly associated with the RC complex and is conserved across all oxygenic phototrophs, and the outer antenna, which can substantially differ among organisms (Croce and van Amerongen 2014). The PSII internal antenna is composed of the pigment-binding proteins CP43 and CP47. In PSI, most pigments forming the inner antenna are associated with the proteins PsaA and PsaB, which also bind the RC. In plants, the outer antenna system of PSI and PSII is composed of members of the light-harvesting complex (LHC) multigenic family (Dall’Osto et al. 2015; Pan et al. 2020). These relatively small integral membrane proteins coordinate between 12 and 17 chlorophylls (Chls) and 2 and 4 carotenoid molecules (see Fig. 2 for the structure of LHCII). Some LHCs are specifically associated with one photosystem, such as the LHCIs to PSI and the minor antenna complexes (CP29, CP26, and CP24) to PSII. LHCII, the most abundant of all, can act as an antenna of both photosystems (Wientjes et al. 2013a). These complexes harvest light and transfer the excitation energy to the RC, increasing its absorption cross section. The structure of a PSII supercomplex of vascular plants is also shown in Fig. 2.

The RC can exist in an “open” or “closed” state. Open RCs are ready to accept excitation energy and initiate electron transfer, while in a closed state, the photosynthetic machinery has temporarily reached its maximum capacity and cannot process new excitations. When excitation energy arrives at a closed RC, it may lead to the formation of reactive oxygen species, which can cause photodamage (Pospisil 2016). This risk highlights the importance of photoprotection mechanisms such as nonphotochemical quenching (NPQ), which dissipates excess light energy as heat, protecting the plant from photodamage and ensuring the efficiency and resilience of photosynthesis.

NPQ refers to a group of photoprotective processes that are accompanied by the quenching of Chl fluorescence under high-light conditions. As pointed out by Horton (2014), experimental evidence as early as 1970 had already established that (i) NPQ depends on the *trans*-thylakoid pH gradient, (ii) it results from nonradiative decay, (iii) this decay has a protective function, and (iv) it might involve alterations in pigment organization within the thylakoid membrane (Papageorgiou and Govindjee 1968; Murata 1969; Wraight and Crofts 1970). All these points are by now generally accepted. However, many aspects of NPQ remain unresolved or at least actively debated. It has been proposed that enabling plants to switch off NPQ more rapidly in fluctuating light conditions can enhance biomass production. While this approach has shown promising results in some plants (Kromdijk et al. 2016; De Souza et al. 2022), it has not proven universally effective across species (Garcia-Molina and Leister 2020; Lehretz et al. 2022).

In this review we introduce the components of NPQ, discuss the various proposals regarding their activation and action mechanisms, and highlight knowledge gaps and controversial aspects. NPQ is a widespread photoprotective mechanism present in nearly all organisms performing oxygenic photosynthesis, including cyanobacteria, green algae, diatoms, red algae, mosses, and vascular plants (Niyogi and Truong 2013; Goss and Lepetit 2015; Muzzopappa and Kirilovsky 2020). However, the molecular players involved, potentially even the mechanisms, differ substantially among these groups. In this review, we focus on NPQ in vascular plants, where most of the mechanistic insights have been obtained to date.

## Where is the danger coming from?

**Figure 3.**
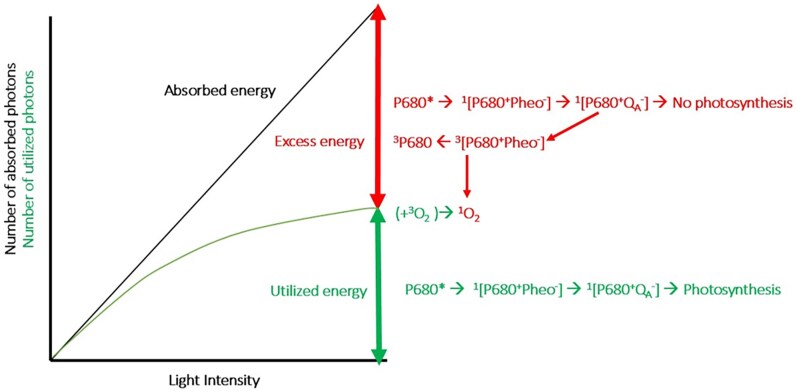
Distribution of absorbed light energy between photosynthesis and other processes. This diagram indicates that the light absorbed by PSII increases linearly with the provided light intensity (black line). However, not all these photons are utilized to drive photosynthesis (green line). At low-light intensities, the fraction of utilized photons is around 87% to 88% (reflected by a difference in slope of 12% to 13%), and at higher intensities, the discrepancy becomes larger due to partial saturation of the photosynthetic process. In that case, a large part of the electrons that have arrived at the quinone $Q_{A}^{-}$ and cannot proceed recombine with the primary electron acceptor Pheo, leading to either the singlet state ^1^[P680 ^+^ Pheo^−^] or a triplet state ^3^[P680 ^+^ Pheo^−^]. This triplet formation in the PSII RC is indicated with the upper red arrow pointing to the left, and such a triplet state can turn oxygen (i.e. in a triplet state in the ground state) into dangerous singlet oxygen. NPQ leads to dissipation of excess energy in the form of heat, thereby reducing the amount of singlet oxygen formation.

Under high-light conditions, not all absorbed photons can be used for photosynthesis due to saturation of the photosynthetic machinery (Fig. 3). As a consequence, electron transfer from the PSII primary donor P680 to plastoquinone becomes inhibited as plastoquinone becomes reduced. This leads to charge recombination from the primary acceptor, a Pheo *a* molecule, back to P680 with a high probability of triplet formation on P680 (^3^P680; Vass 2011; Telfer 2014). Such a triplet can easily react with molecular oxygen to generate singlet oxygen (^1^O_2_), which is highly reactive and can lead to severe damage.

Alternatively, charge recombination can result in the formation of singlet excitations diffusing back into the PSII antenna, potentially leading to triplet formation via intersystem crossing on one of the antenna Chl *a* pigments. These triplets are usually rapidly quenched by nearby carotenoids, which greatly reduces the probability of ^1^O_2_ formation (Siefermann-Harms 1987). However, it is important to note that only a fraction of recombination events generates excited singlet states and thus forms triplets via intersystem crossing. Most triplets are formed via recombination directly on P680 (De Grooth and van Gorkom 1981; Krieger-Liszkay and Rutherford 1998; Rappaport et al. 2002). Regardless of the exact recombination pathway, protection is needed to minimize the formation of ^1^O_2_ or other reactive oxygen species. NPQ serves this protective function by shortening the excited-state lifetime in PSII, reducing the number of charge separation events in its RC, and thereby lowering the probability of ^1^O_2_ formation. Indeed, experiments with Arabidopsis and rice mutants have shown that NPQ confers a strong fitness advantage under field conditions and is particularly important in increasing plant tolerance to fluctuating light (Muller et al. 2001; Kulheim et al. 2002; Ikeuchi et al. 2014; Steen et al. 2020).

## What general strategies can be used to protect PSII against overexcitation?

Maintaining a balance between PSII light absorption and the use of the resulting electrons in the linear electron transport chain is essential to prevent photodamage. When PSII becomes overexcited, alternative electron sinks can help limit reactive oxygen species production (see Alric and Johnson 2017 for a review), but here we focus on the strategies that directly reduce excitation pressure on the PSII RC to keep reactive oxygen species production within “acceptable limits.” This can be achieved by reducing the light-harvesting (LH) capacity of PSII, which is the product of the absorption cross section (σ_abs_) and the quantum efficiency of charge separation (ϕ_cs_) in the RC (van Amerongen and Wientjes 2022). The LH capacity is presumably maximized in low-light conditions to capture as much light as possible, but in high light, it can be reduced by decreasing σ_abs_ and/or ϕ_cs_. Reducing the absorption cross section can be done at the macroscopic level via relatively slow processes such as leaf movement, leaf curling, and leaf deposits; at the cellular level via chloroplast movement (Ruban et al. 2012; Demmig-Adams et al. 2014); and at the molecular level via antenna reduction (slow) or detachment (potentially fast) from the photosystem. For instance, the antenna size is reduced in plants grown at high light (Anderson and Andersson 1988; Ballottari et al. 2007; Wientjes et al. 2013b), and even within one leaf, the antenna size at the adaxial side can be significantly smaller than on the abaxial side (Iermak et al. 2016). Detachment of part of the antenna, which can happen on a much faster time scale, has been reported for plants (Betterle et al. 2009; Holzwarth et al. 2009). Alternatively, the LH capacity can be lowered by reducing ϕ_cs_ by introducing 1 or more quenchers in the PSII antenna, which decrease the excited-state lifetime, thereby reducing the probability that the excitation energy reaches the RC. This is probably the fastest possible response to changes in light intensity, and it is, in fact, the operating principle of NPQ.

## How is NPQ measured?

**Figure 4.**
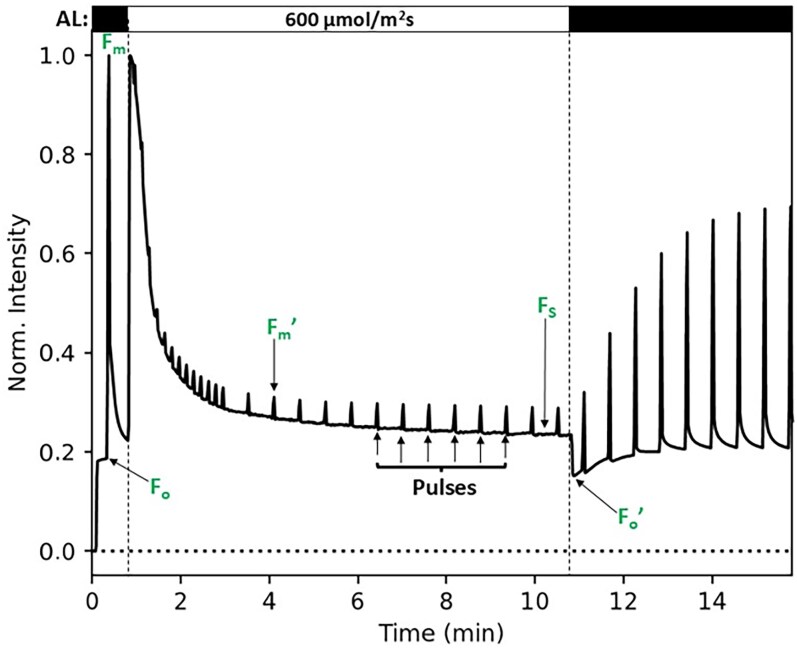
Fluorescence of *A. thaliana* leaf measured with PAM fluorometry as a function of time at different light conditions. Initially, the amount of fluorescence is recorded from a dark-adapted leaf (*F_o_*) in which all PSII RCs are open (black bar on the top to the left); this is followed by measurement of fluorescence after a high-intensity saturating light flash (*F_m_*) that is supposed to close all RCs. Subsequently, during illumination with actinic light of 600 μmol m^−2^·s^−1^ (top bar, white part in the middle), the basic fluorescence (*F_s_*) goes down due to NPQ, and so does the fluorescence intensity after the application of saturating flashes during actinic illumination $\left({F^{\prime }}_{m}\right)$. The amount of quenching or NPQ is usually defined as $\mathrm{NPQ}=(F_{m}-{F^{\prime }}_{m})/F_{m}^{\prime }=F_{m}/F_{m}^{\prime }\text{–}1$. Its value is around 3.3 after 10 min in this particular experiment. Immediately after the actinic light is switched off, $F_{o}^{\prime }$ can be measured, reflecting the fluorescence in the presence of open RCs but also in the presence of NPQ.

NPQ reveals itself as a reduction of fluorescence of leaves or cells upon light exposure, and it is often measured by PAM (pulse amplitude modulation) fluorometry. The fluorescence is initially recorded from a dark-adapted leaf (*F_o_*) in which all PSII RCs are open; this is followed by measurements of fluorescence after a high-intensity saturating light flash (*F_m_*), which is assumed to close all RCs (Fig. 4). When the leaves are subsequently illuminated with high actinic light intensities, the basic fluorescence (*F_s_*) goes down due to NPQ, and so does the fluorescence intensity after the application of saturating flashes during actinic illumination $\left({F^{\prime }}_{m}\right)$. The amount of quenching or NPQ is usually defined as $\mathrm{NPQ}=(F_{m}-{F^{\prime }}_{m})/F_{m}^{\prime }=F_{m}/F_{m}^{\prime }\text{–}1$.

If we assume that the Butler model applies (Appendix; Butler 1978), it can be shown that NPQ = *k*_NPQ_/(*k_F_* + *k*_IC_ + *k*_ISC_). The rate constants are all depopulation rates for the excited state, and together they determine the excited-state lifetime: *k*_NPQ_ is the rate of NPQ; *k_F_* is the radiative rate or rate of fluorescence; *k*_IC_ is the rate of internal conversion or heat formation, not including NPQ; and *k*_ISC_ is the rate of intersystem crossing or triplet formation, which typically results in heat dissipation. Instead of determining *k*_NPQ_ with closed RCs, as is most commonly done in the literature, it is possible to obtain *k*_NPQ_ with open RCs. Although it is generally assumed that the *k*_NPQ_ rate is identical for open and closed RCs, it has been reported for spinach that *k*_NPQ_ can be significantly smaller in the case of open RCs (Farooq et al. 2018; van Amerongen and Chmeliov 2019), although this finding has yet to be confirmed by other researchers.

## Does NPQ also occur in PSI?

NPQ is measured as a decrease in PSII fluorescence, and as previously noted, this is the photosystem that needs protection. However, it has been suggested that PSI might also be a site of NPQ in plants (Ballottari et al. 2014; Sapeta et al. 2023), mosses (Pinnola et al. 2015), and green algae (Girolomoni et al. 2019; Girolomoni et al. 2020). These suggestions are primarily based on 77-K fluorescence emission data of cells and membranes, where a 20%–25% decrease in the intensity of the red-most emission peak (attributed to PSI) was observed with changes in excited-state lifetimes. Yet, interpreting these changes requires caution. At 77 K, the excited-state lifetime of PSI is long (∼2 ns) since the far-red Chls act as excitation traps at this temperature (Croce et al. 2000). The observed changes of 20% to 25% in the fluorescence of the far-red PSI peak would then correspond to an inverse quenching rate of only 6 to 8 ns. By contrast, the overall trapping time of PSI under physiological temperature is in the range of 50 to 100 ps, meaning that such a slow process would quench <1% of the excited states. This is in agreement with experimental observations showing no differences in PSI excited-state lifetimes in quenched and unquenched conditions at room temperature (Tian et al. 2017; Sapeta et al. 2023).

Additionally, it is important to consider that the far-red fluorescence peak of the 77 K includes a contribution from PSII. This complicates the attribution of spectral changes solely to PSI quenching. In fact, PSII emission >700 nm is a well-known feature exploited in PAM fluorometry, where changes in PSII fluorescence are measured at wavelengths >700 nm. Although PSI is the main emitter in this spectral region at low temperatures, the presence of PSII emission and the reduction of its emission due to NPQ may explain the observed changes in the 77-K spectra.

In conclusion, NPQ of PSI at room temperature is negligible (i.e. <1%).

### Does PSI need to be quenched?

The excited-state lifetime of PSI-LHCI in plants is approximately 50 to 100 ps and remains essentially unchanged regardless of the redox state of P700, the primary electron donor in PSI. This phenomenon occurs because the oxidized form, P700+, acts as a highly effective excitation quencher (Savikhin 2006). The quenching rate of P700+ is far higher than the quenching rates observed for PSII during NPQ across various organisms (Wientjes and Croce 2012). As a result, overexcitation of PSI is rarely a concern. In fact P700+ has a protective role, especially under conditions where PSI electron acceptors are limiting and the FeS centers become overreduced, increasing the risk of oxidative stress and PSI damage (Sonoike 2011). Therefore, the optimal strategy to prevent PSI photodamage is to limit electron transfer from PSII, thereby generating P700+. This protective state can be achieved via quenching in PSII and photosynthetic control at the cytochrome b_6_f (cyt b_6_f) or alternative electron transfer pathways (Foyer et al. 2012; Suorsa et al. 2012; Storti et al. 2020; Lima-Melo et al. 2021). In this way, NPQ of PSII can also indirectly protect PSI.

## Which processes are collectively called NPQ?

As described earlier, NPQ is measured as a decrease in PSII fluorescence. However, this decrease results from multiple processes operating on different time scales, ranging from seconds to minutes, with different dependencies on light intensity.

The major and fastest component of NPQ is qE, or energy-dependent quenching, which develops within tens of seconds (Wraight and Crofts 1970; Krause and Weis 1991) and is supposedly triggered by the proton gradient (ΔpH) across the thylakoid membrane (Wraight and Crofts 1970; Krause 1974; Horton and Ruban 1992; Horton et al. 1996). qE reduces excitation energy transfer (EET) to PSII RCs. It requires the presence of the thylakoid membrane integral protein PsbS (Li et al. 2000), and it is enhanced by the xanthophyll cycle: the low pH that is created in the lumen in high-light conditions activates the enzyme VDE (violaxanthin de-epoxidase), which causes de-epoxidation of violaxanthin (Vx) to zeaxanthin (Zx) in a 2-step process via the formation of the intermediate antheraxanthin (Yamamoto et al. 1962; Demmig et al. 1987). Zx accumulation leads to increased levels of NPQ but on a slower time scale (several minutes). Some authors include this Zx-dependent phase within qE (Ruban et al. 2012; Horton 2014), while others distinguish it as qZ (Dall’Osto et al. 2005; Nilkens et al. 2010).

While PsbS- and Zx-dependent mechanisms clearly belong to NPQ, other processes can reduce fluorescence but do not contribute to NPQ. One such phenomenon is chloroplast movement. Under high-light conditions, chloroplasts rearrange within cells, lining up behind each other to minimize light absorption through shading, thereby lowering fluorescence (see e.g. Davis et al. 2011). This effect can already be observed after 10 min and may take between 30 min and a few hours, depending on species and light intensity. It is triggered by blue light via the phototropin photoreceptor and does not occur under red light (Kagawa et al. 2001). Experimentally, apparent NPQ due to chloroplast movement can be distinguished from true NPQ by picosecond fluorescence measurements because movement will lead to a decrease of the amplitudes of the time-resolved components, whereas quenching will lead to a shortening of the excited-state lifetimes (Amarnath et al. 2012; Farooq et al. 2018; Park et al. 2019).

Another process that can decrease fluorescence is state transitions (sometimes called qT), during which part of the LH antenna (some LHCII trimers) shuttles between PSI and PSII to optimize excitation balance when the available light is more selective for one of these photosystems (Goldschmidt-Clermont and Bassi 2015). Since LHCII leads to less fluorescence when connected to PSI than to PSII, the movement toward PSI looks like quenching, while it is in fact due to decreased PSII absorption.

The most slowly forming and relaxing NPQ component is called qI, or sustained quenching, which is ascribed to the photoinhibitory damage of PSII RCs that persists for hours in the dark (Krause 1988). Another quenching process, called qH, has more recently been proposed, but it appears to be active only at low temperatures, and we refer to Malnoë (2018) for more details. In this review, we focus on qE, the fast component of NPQ, which, according to our definition, includes PsbS- and Zx-dependent quenching in plants.

## Where does the quenching occur?

The answer to this question has differed quite a lot over the years, and the minor LH antennas (CP24, CP26, and CP29) and major LH antenna (trimeric LHCII) have all been proposed as major quenching sites (Avenson et al. 2008; Holzwarth et al. 2009; Ruban 2016; Dall’Osto et al. 2017) as well as the PSII core complex (Ivanov et al. 2008). The first proposal that LHCII was the primary site of quenching arose from the observation that aggregated LHCII complexes are highly quenched (Horton et al. 1991; Ruban and Horton 1992). However, quenching upon aggregation later appeared to be a more general property of pigment–protein complexes independent of the type of complexes and the organism of origin (Mascoli et al. 2020; Ueno et al. 2025). By contrast, the initial hypothesis that CP29 and CP26 could serve as quenching sites was based on their higher Vx content, leading to the suggestion that in high light these proteins act as the main binding sites for Zx (Bassi et al. 1993). This hypothesis was supported by the presence of protonatable residues that can act as pH sensors in their structure, while this is not the case in LHCII (Walters et al. 1994; Pesaresi et al. 1997). Moreover, both proteins, particularly CP29, have long been considered attractive candidates for quenching because of their strategic position between LHCII and the PSII core (Yakushevska et al. 2003), where they could function as a gateway for EET. Despite these arguments positioning the minor antenna complexes as ideal hubs for quenching (Caffarri et al. 2011; Yang et al. 2024), experimental evidence does not support this hypothesis: (i) Zx was not found to be bound to the minor antenna complexes under high-light conditions (Xu et al. 2015); (ii) antisense mutants lacking CP29 or CP26 do not exhibit significant changes in overall NPQ levels (Andersson et al. 2001). While mutants without CP29 display a slower NPQ induction, they ultimately reach wild type (WT) NPQ levels (de Bianchi et al. 2011). Given that the loss of CP29 alters the energy transfer between the external antenna and the PSII core (van Oort et al. 2010), the delayed kinetics likely reflects changes in the supercomplex organization rather than direct involvement of CP29 in quenching. A recent comprehensive in vivo study also excluded CP26 as the site of qE and qZ (Walter et al. 2024). Similarly, although CP24 knockouts show reduced quenching, double mutants lacking CP24 and CP26 restore NPQ to WT levels, suggesting that the initial effect arises from changes in membrane organization rather than from direct involvement in quenching (de Bianchi et al. 2008). Plants lacking all minor antenna complexes exhibit slower NPQ induction kinetics but reach the same maximal NPQ as WT (Dall’Osto et al. 2017). Studies using these mutants pointed toward LHCII as the principal site of quenching, arguing against a central role for the minor antenna proteins (Townsend et al. 2018).

Mutants of *Arabidopsis thaliana* lacking Lhcb1 (Pietrzykowska et al. 2014), especially mutants completely lacking the LHCII trimers (Nicol et al. 2019), instead show a strong reduction in NPQ, leading to the conclusion that most of the quenching is associated with this complex. However, while the majority of the quenching occurs in LHCII, some quenching can still happen in the absence of the main LHCII subunits Lhcb1 and Lhcb2 and even when the minor antennae are missing. These findings suggest that, in addition to LHCII trimers, Lhcb3 and/or minor antenna complexes, as well as the PSII core, can contribute to NPQ, albeit to varying extents (Nicol et al. 2019).

In summary, we believe that all complexes have the capacity to quench, but the majority of the quenching occurs in LHCII.

## How can LHCs also function as quenchers?

The primary role of the LHCs is to harvest light and efficiently transfer excitation energy to the RC, seemingly the opposite of energy quenching. So, how can a pigment–protein complex designed for LH become a quenching site? The prevailing view is that during NPQ, the LHCs undergo a conformational change that modifies the interactions between the pigments (e.g. by changing their distances or orientations), thus creating a quenching center. It has long been recognized that the LHCs adopt multiple conformations, each with distinct excited-state lifetimes (Moya et al. 2001; van Oort et al. 2007b) and spectra (Kruger et al. 2012), supporting this conformational switch model. van Oort et al. (2007b) showed that a change in hydrostatic pressure could shift the equilibrium between unquenched (3.5 to 4 ns) and quenched conformations, with the latter showing two lifetimes: one that is ∼0.5 ns and one as short as ∼25 ps. The 25 ps represents a highly efficient quenching state, where energy is rapidly trapped and the measured lifetime is dominated by excitation migration to the quencher (van Amerongen and van Grondelle 2001).

In contrast, the ∼0.5-ns lifetime indicates slower quenching. Notably, a difference of 0.006% in the volume of trimeric LHCII for the 0.5- and 3.5-ns forms was measured, suggesting that only subtle structural changes are involved. Interestingly, the 0.5-ns quenched state was accompanied by a relative increase of an absorption band at 505 nm, which hints at a conformational change of a carotenoid. Although the 25-ps state is too strongly quenched to allow detection of corresponding spectral changes, it is plausible that a carotenoid transition also occurs in this state. A similar change in absorption is observed in vivo upon induction of NPQ and is often ascribed to the formation of Zx (Bilger et al. 1989; Johnson et al. 2009). Yet in the van Oort study, no VDE was present (van Oort et al. 2007b), indicating that Zx formation is not required for this signal. Whether spectral changes correspond to the same quenching mechanisms active during NPQ in vivo remains an open question.

## Identikit of the quencher/quenchers

For a molecule to serve as an efficient quencher of Chl singlet excited states, several requirements must be met. First, it must be physically close or part of the pigment–protein complexes mentioned earlier. Second, its excited-state energy must be close to that of the first excited state of Chl *a*, which allows it to accept excitation energy from Chl *a* molecules via EET or excitonic coupling. The process must also occur on a subnanosecond timescale to outcompete the other decay processes. Efficient quenchers typically have extended π-conjugated systems, such as Chls or carotenoids. Alternatively, quenching can occur via electron transfer to or from an excited Chl, provided that the quencher has suitable redox properties. Quinones, for example, are well-known quenchers of Chl fluorescence through this mechanism (Amesz and Fork 1967).

### Are carotenoids the quenchers?

Carotenoids seem to have all the prerequisites for acting as quenchers: they are components of the photosynthetic complexes, they have excited-state levels close to the lowest excited (singlet) state of Chl *a*, and their excited-state lifetimes are very short (i.e. on the order of 10 ps; Fig. 4). Various proposals involving carotenoids regarding the mechanisms of NPQ have been put forward, making it challenging to navigate the literature. To provide a comprehensive explanation of the proposed mechanisms, including ones that have been discarded and why, we present here a short historical overview of this topic.

### A historical overview regarding carotenoid quenching

#### Gear shift model

The molecular gear shift model of Frank et al. (1994) was the first in a row of many molecular models that involved a xanthophyll as a quencher. As mentioned previously, at low lumenal pH, Vx is converted to Zx, and because of the increase in the number of double bonds in the conjugated chain, the S_1_ energy level was thought to decrease from above to below that of the Chl Q_y_ energy. Therefore, when Vx in the LHCs would be replaced by Zx, the S_1_ state would turn from a donor into an acceptor of excitation energy via an incoherent energy transfer mechanism. However, other studies showed that the energy levels are different than expected (Polivka et al. 1999; Frank et al. 2000), and this was one of the reasons that the gear shift model was abandoned (for reviews, see Horton 2014; Polivka and Frank 2014).

#### Excitonic Chl–Car interaction, part I

Another quenching mechanism was proposed by van Amerongen and van Grondelle (2001), who argued that relatively strong coupling between the S_1_ state of lutein (Lut) and the Q_y_ state of Chl *a*, as observed by fs transient absorption measurements, would lead to a shared exciton state between the molecules, thereby mixing the fast excited-state decay rate of Lut, which is (∼10 ps)^−1^, with the much slower one of Chl *a* (decay time of several ns). However, while this mixing is unavoidable, the extent to which it can be regulated was not clear. This model lived a somewhat dormant existence for many years until it was revived by Walla and coworkers (Bode et al. 2009) (see section Excitonic Chl-Car interaction, part II).

#### Chl^+^-Z^−^ radical cation formation

In 2003, Zx entered the scene again as the proposed quencher but now as part of a Chl-Zx heterodimer that undergoes charge separation upon excitation, leading to Zx cation formation (Dreuw et al. 2003). This theoretical proposal was supported by femtosecond pump–probe measurements on thylakoid membranes (Holt et al. 2005). Later studies suggested that Zx cations could also be induced in the minor antenna complexes CP29, CP26, and CP24 and not in LHCII, but the amount of cation formation was extremely small (Avenson et al. 2008). It was suggested that Zx binds into the internal carotenoid binding site L2 in these complexes and forms a quencher in association with Chl a603 (nomenclature from Liu et al. 2004) (Ahn et al. 2008). However, it was later shown that no Zx is present in the L2 site in vivo in physiological conditions (Xu et al. 2015). Moreover, whether in LHCII or minor antennae, the presence of the Zx radical cation has not yet been found to be associated with any significant quenching of Chl fluorescence as compared with Vx-containing complexes (Amarie et al. 2007; Ahn et al. 2008; Amarie et al. 2009). Whether the Zx radical cation is a more efficient quencher in vivo via interaction with other factors, as suggested by Ahn et al. (2008), remains to be demonstrated.

#### Chl-Zx S_1_ EET

Although the gear shift model was abandoned, it does not mean that Zx may not be directly involved in quenching. For instance, the Fleming group proposed, on the basis of femtosecond transient absorption measurements on thylakoids in an NPQ state, that EET occurred from Chls to Zx (Ma et al. 2003). But shortly after, given new femtosecond transient absorption measurements, the group proposed the Chl^+^-Z^−^ radical cation quenching model (see previous paragraph), although it was recently proposed again that the Zx S_1_ state could act as a direct quencher (Lee et al. 2024). We address this proposal in the “Where is Zx located?” section where we argue why we do not consider it very likely that such a process occurs in WT plants.

#### Chl-Lut1 S_1_ EET

A femtosecond pump–probe study concluded that quenching is caused by EET from a cluster of low-energy Chl *a* molecules (611 and 612) to Lut L1 (620) in LHCII (Ruban et al. 2007). This LHCII was brought to the dissipative state by oligomerization, which led to the same characteristic Raman signature seen in quenched LHCII crystals (Pascal et al. 2005) and in leaves in NPQ conditions. However, the study by Ruban et al. (2007) had some critical limitations. Although singlet–singlet annihilation was known to be present, its potential contribution to the bleaching signal attributed to L1 was not considered. Later work by van Oort et al. (2018) demonstrated that the L1 signal could result from the high-intensity laser excitation, casting doubt on the original interpretation. So, while L1 remains a plausible quencher, the most compelling experimental evidence for this had disappeared again. Moreover, although earlier theoretical models supported this mechanism (Chmeliov et al. 2015; Balevicius et al. 2017; Fox et al. 2017, 2018), more recently, Duffy and collaborators (Gray et al. 2022) concluded that EET to S_1_ is unlikely to provide the quenching mechanism since the pigment rearrangements are too limited to switch LHCII from a LH to a quenched state. The authors also concluded that the modulation of the S_1_ energy level is unlikely to be at the origin of the quenching switch.

#### Excitonic Chl–Car interaction, part II

In the meantime, a study by Walla and coworkers (Bode et al. 2009) provided support for the earlier proposal by van Amerongen and van Grondelle (2001). Using 2-photon absorption spectroscopy to selectively excite the carotenoid S1 state in LHCII, the authors observed a close correlation between Chl–Car interaction strength and NPQ levels. Follow-up studies from the same group (Liao et al. 2010) reinforced this conclusion (see Holleboom and Walla 2014 for an overview) and extended it to CP24 and CP29 (Holleboom et al. 2015). However, Betke and Lokstein (2019) argued that the authors had incorrectly assumed that they excited carotenoids only with their experimental technique, whereas the amount of absorption by Chls was reported to be even higher. Moreover, it was still unclear how Chl–Car coupling strength could strongly increase upon the transition from unquenched to quenched states, especially since theoretical studies have argued that there is hardly any change in interaction strength upon structural changes (Gray et al. 2022; Accomasso et al. 2024).

#### Chl-Lut1* EET

**Figure 5.**
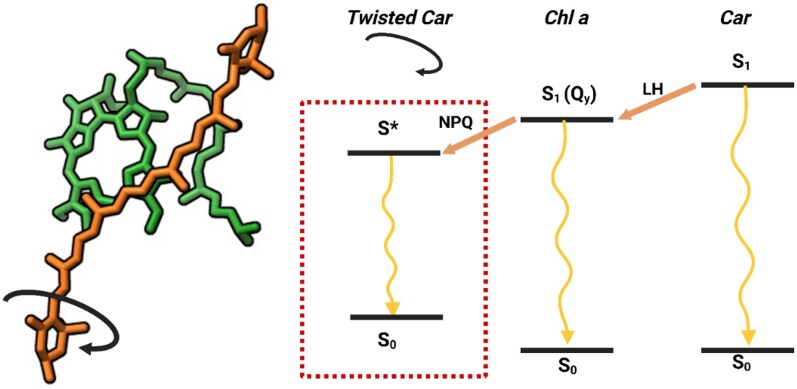
Proposed model for excitation energy transfer between carotenoids (cars) and chlorophylls (chls) in LH and in photoprotection (NPQ) conditions. Schematic energy diagram of the lowest electronic states of a Chl *a* and a Car molecule—that is, the ground state S_0_ and the first excited state of all molecules. The first excited-state S_1_ Car on the right corresponds to the situation that all antenna complexes are in the LH state, and excitation energy is rapidly transferred unidirectionally from Car to Chl *a*. When NPQ occurs in this particular example, the Car is in a twisted conformation (Car on the left); this leads to a lowering of the first excited state, which becomes an S* state that can accept excitations from a neighboring Chl. This process is almost unidirectional and is followed by rapid decay to the ground state of the twisted carotenoid, typically within 10 ps.

In 2017, Liguori et al. (2017) used femtosecond transient absorption spectroscopy on quenched monomeric LHCII in which all xanthophylls were replaced by astaxanthin, and they concluded that quenching occurred via EET from Chls to a carotenoid state distinct from S_1_. This state resembled the so-called S* state, proposed to be either an excited state of a distorted carotenoid (Gradinaru et al. 2001; Niedzwiedzki et al. 2006) or a hot ground state (Buckup et al. 2006). Subsequent work supported this idea. Mascoli et al. (2019), studying isolated CP29, found that a fraction of the complexes was quenched, and the spectral feature of the quencher (absorption around 505 nm) pointed to a carotenoid S*-like state. Similar results on CP29 were later reported by Sardar et al. (2022). Liguori et al. (2017) proposed that the switch from LH to a quenching state could involve the rotation of a carotenoid ring, which made EET to the S*-like state partially allowed. It is of interest to mention that isolated LHCII trimers develop quenching under high hydrostatic pressure, and this is accompanied by the induction of a 505-nm absorption band that can be observed in the fluorescence excitation spectrum (van Oort et al. 2007b). Presumably, the exact nature of the carotenoid is not that important. However, it is interesting to note that while quenching in vivo is induced when LHCII contains Lut, Zx, and even astaxanthin, this is not the case when the internal binding sites are occupied by Vx as in the npq1lut2 mutant (Niyogi et al. 2001). This is particularly notable because among the xanthophylls, Vx has been shown to have the lowest yield of S* formation and the longest excited-state lifetime (Niedzwiedzki et al. 2006). By using quantum chemical and molecular dynamics simulations, Accomasso et al. (2024) recently identified a minor s-*trans* conformer of Lut in CP29 that could act as a quencher. This conformer showed a shorter excited-state lifetime and a blue-shifted excited-state absorption as compared with the dominant s-*cis* conformer. They proposed that this species corresponds to the experimentally observed quencher in CP29, in particular because of the broad S_1_ energy distribution, which spans a few eV and includes values lower than Chl Q_y_ states. Yet, they did not observe changes in coupling strength between Lut and nearby Chls, and it has not been quantitatively demonstrated that the calculated quenching rate is sufficiently large to explain the experimentally observed NPQ. A schematic overview of the proposed mechanism is given in Fig. 5.

Finally, we want to emphasize that nearly all the studies of the molecular mechanisms of NPQ were performed with ultrafast transient absorption measurements in vitro, which can be extremely useful to trace potential ways in which quenching is functioning at the molecular scale. Still, they do not necessarily provide the correct in vivo mechanism of NPQ. Measurements were also performed on thylakoids, which, although more representative of the in vivo system, present several challenges, including the difficulty of inducing NPQ in isolated membranes (Sacharz et al. 2017) and potential changes in membrane organization that may occur during the measurements (Sznee et al. 2011). Validation of the proposed mechanisms in vivo, however, is usually extremely difficult, among others, because of signal distortion due to light scattering in the leaves and might be possible only by coupling targeted mutational analysis and physiological measurements. Yet, pleiotropic effects of the mutations and different levels of protein expression might still influence the results in that case. A preparation of microsized cell fractions from the green alga *Chlamydomonas reinhardtii* was recently shown to enable transient absorption measurements of NPQ (Zheng et al. 2024). This strategy might help to bridge the gap between in vitro and in vivo studies, but it remains to be seen whether this method would be better than studying thylakoid membranes isolated from plants.

### Can Chl–Chl charge transfer states act as quenchers?

An alternative quenching mechanism independent of carotenoids involves the formation of charge transfer states between Chls (Miloslavina et al. 2008; Pawlak et al. 2020). The proposal of this mechanism was originally based on a far-red fluorescence feature observed for LHCII oligomers and Arabidopsis leaves under NPQ conditions (Miloslavina et al. 2008). However, the intensity and spectral shape of the red-shifted emission vary widely across LHCII oligomer samples. Among these, only one showed a spectrum resembling the 400-ps decay-associated spectrum that was supposed to reflect NPQ. Follow-up studies (Miloslavina et al. 2011) comparing time-resolved fluorescence in WT and mutant Arabidopsis revealed large variability in the supposed red-shifted oligomeric LHCII band. In contrast, completely different spectra of quenched leaves were reported by Farooq et al. (2018), further questioning the general relevance of this feature. Moreover, Passarini et al. (2010) demonstrated that the red-shifted forms of isolated LHCs are associated with long excited-state lifetimes, the opposite of the short-lived states required for efficient quenching in NPQ. Chmeliov et al. (2016) later reported that red-shifted forms are indeed present in aggregated LHCII and were ascribed to mixing of excitonic and charge transfer states based on the similarity with Lhca emission (Romero et al. 2009). Yet, these states are visible only at cryogenic temperatures, where they act as energy traps and were shown not to be directly involved in quenching (Chmeliov et al. 2016; Gelzinis et al. 2018). The role of aggregation is discussed later.

In summary, if low-energy states are present or can be created in plants, their involvement in quenching remains speculative, as there is currently no strong evidence supporting such a role and available data suggest that they are unlikely to play a major part.

### Is more than one quenching mechanism active in the membrane?

As several quenching mechanisms have been proposed based on the experiments discussed earlier, it may seem plausible that several of them could operate simultaneously in the thylakoid membrane. For example, Holzwarth et al. (2009) proposed, partly on the basis of time-resolved fluorescence measurements, that two distinct quenching processes occur: one localized in clustered LHCII and dependent on PsbS and one in the minor antenna complexes that is Zx dependent (the validity of this model is discussed in the section Is there structural/biochemical evidence for reorganization/aggregation of photosynthetic complexes during NPQ). More recently, Lee et al. (2024) argued that at least two quenching mechanisms coexist in the thylakoid membrane: one involving quenching by Lut and one by Zx (for further discussion see section **where is Zx located?**). Also, Ramakers et al. (2025a) reported two mechanisms acting on different time scales. However, when considering the coexistence of different quenching mechanisms, it should be realized that qE is almost completely absent without protonated PsbS (Li et al. 2000, 2004) and thus all mechanisms must be PsbS dependent.

## How is qE activated?

Although antenna complexes can adopt multiple conformations with distinct lifetimes and spectral properties switching between them in vitro, such spontaneous transitions would be detrimental in vivo. In living organisms, the transition from an LH to a quenched conformation must therefore be carefully regulated. But how is this switching controlled?

It has long been established that in plants the trigger for qE is the pH difference (ΔpH) across the thylakoid membrane, which is particularly large in high-light conditions (Krause and Behrend 1986; Krause et al. 1988; Rees et al. 1992; Noctor et al. 1993). During photosynthesis, protons accumulate in the lumen, lowering its pH relative to the stroma. This acidification correlates with excitation pressure and activates qE. Although ΔpH and low lumenal pH are coupled in vivo, studies have shown that acidifying the lumen alone is sufficient to induce qE (Tian et al. 2019; Pang et al. 2023). This identifies low pH as the key trigger for activating the protective quenching response, rather than the ΔpH.

### What are the direct molecular consequences of the low lumenal pH?

Low pH of the thylakoid lumen is thought to induce the protonation of LHCII, the PsbS protein, and VDE, with the latter catalyzing the conversion of Vx to Zx. These 3 events are widely believed to be essential steps in NPQ induction and are discussed individually.

## Does the low lumenal pH directly trigger quenching in LHCII?

An early hypothesis suggested that protonation of lumen-exposed glutamate and aspartate residues of LHCII could drive a conformational change leading to LHCII aggregation, concomitantly switching LHCII from a LH to an energy-dissipative state (Ruban et al. 1992a, 1992b, 1994a, 1994b, 1996; Horton et al. 1996; Phillip et al. 1996). More recently, Ruan et al. (2023) reported the cryo-EM structures of LHCII at pH 7.8 and 5.4 in nanodiscs and detergent. They observed a local structural difference attributed to protonation of D54 and E207, but time-resolved fluorescence measurements in the same study showed no difference in fluorescence decay kinetics between the pH conditions for trimeric LHCII (for extended data, see Fig. 2 of the same article). These results are consistent with earlier measurements showing that low pH alone does not induce quenching of LHCII in detergent (Liguori et al. 2013) or in liposomes (Nicol and Croce 2021) and with the fact that in the Arabidopsis npq4 mutant, which lacks PsbS, qE is not induced in natural light (Li et al. 2000). Quenching was instead observed at low pH in liposomes containing PsbS and LHCII (Nicol and Croce 2021). Given these results, it was proposed that low pH induces a structural rearrangement in LHCII that creates a poised prequenching state. This conformation may not be sufficient to trigger energy dissipation on its own but could be pushed into a fully quenched state through interaction with PsbS and/or Zx. Whether LHCII aggregation is involved in this process is addressed in the following section.

## Role of PsbS in NPQ

PsbS is widely recognized as the central regulator of NPQ in plants, but it probably also remains the greatest enigma, as its exact role and action mechanism remain incompletely understood. Its importance was first established by Niyogi and coworkers, who demonstrated that mutants lacking PsbS were unable to induce substantial NPQ in natural light conditions (Li et al. 2000, 2002b), while overexpression led to enhanced quenching (Li et al. 2002c). But what is its role? Different proposals have been made, and they are presented in turn with some of the molecular properties of PsbS (see Marulanda Valencia and Pandit 2024 for a recent review on PsbS).

### Does PsbS bind pigments?

PsbS is a member of the LHC multigenic family (Niyogi and Truong 2013) and contains 4 transmembrane helices, 2 of which are highly homologous to the central helices of the LHCs (Fan et al. 2015). However, unlike canonical LHCs, PsbS does not bind pigments. Although some conserved Chl-binding residues are present, even after mild solubilization, no pigments appear to be bound, and the protein can also be reconstituted in vitro in the absence of Chls (Dominici et al. 2002). Does it maybe bind carotenoids? Bonente et al. (2008) specifically addressed the binding of xanthophylls and concluded that no xanthophylls are stably bound. This finding also seems to exclude that PsbS acts directly as a quencher. Yet, the possibility of transient pigment binding cannot be ruled out. For instance, PsbS might bind Zx when it interacts with other complexes, potentially contributing to the formation of quenching centers. Nevertheless, there are currently no experimental data supporting this hypothesis.

### Is PsbS a pH sensor?

One well-established PsbS function is its role as a pH sensor. In mutants where 2 lumen-exposed glutamates are substituted with nonprotonatable residues, NPQ fails to activate despite normal PsbS assembly, which indicates that PsbS activation occurs via protonation (Li et al. 2004). Glutamate typically has a pKa value of 4, which seems too low to respond to the thylakoid lumenal pH, where the pH does not usually drop below 5.5, as lower values would damage the water-splitting catalysts associated with PSII (Kramer et al. 1999) and inhibit electron transport at the cyt b_6_f complex (Tikhonov 2013). However, MD simulations have shown that the local protein environment modulates the pKa of the residues, resulting in most of the lumen-exposed Glu having a pKa value >5.5 (Liguori et al. 2019), making them sensitive to physiological pH in the lumen.

### PsbS: dimer or monomer?

A recurring claim is that PsbS is dimeric in its inactive state at neutral pH and monomerizes upon protonation at low pH, becoming active. This idea originates from SDS-PAGE experiments, where PsbS appears dimeric at pH 7 and monomeric at pH 5 (Bergantino et al. 2003). However, SDS-PAGE is performed in denaturing conditions, not in the protein's native state, and the protein may remain dimeric during denaturation due to strong hydrophobic, ionic interactions or hydrogen bonds. PsbS is a very hydrophobic protein; at low pH, protonation of acidic residues reduces surface charge and further increases hydrophobicity. This is in line with the crystal structure that shows a dimeric complex at pH 5 (Fan et al. 2015), contradicting the idea of pH-induced monomerization. In addition, experimental (Krishnan-Schmieden et al. 2021) and computational (Liguori et al. 2019; Chiariello et al. 2023) studies have demonstrated that the unprotonated form has weaker intersubunit interactions due to the destruction of H-bonds at the dimer interface, not the other way around. Altogether, these results call into question the monomer/dimer model. Instead, it has been proposed that the low pH strengthens PsbS's ability to interact with LHCs, possibly forming heterodimers, in which PsbS interacts with the LHCs, thus stabilizing their quenched state (Liguori et al. 2019).

### Where is PsbS located? And what are its interacting partners?

PsbS has not been visualized in any cryo-EM structure of PSII supercomplexes, although in sucrose gradients its presence was observed in practically all bands (Caffarri et al. 2009), and a pull-down assay indicated that PsbS interacts with most of the photosynthetic proteins (Teardo et al. 2007). One of the reasons for those many interactions might be the hydrophobicity, which could mediate nonspecific associations during purification. Cross-linking experiments on thylakoid preparations have shown interactions with LHCII and minor antennae (Sacharz et al. 2017), PSII core components (Correa-Galvis et al. 2016), and even PSI components (Gerotto et al. 2015). Notably, enhanced interactions with Lhcb1 under high-light conditions, which should correspond to the PsbS active state, have been reported (Correa-Galvis et al. 2016; Sacharz et al. 2017). However, these interactions are not exclusive to the quenched state, and a general increase in interactions of PsbS with all the PSII components in light-treated samples complicates the identification of functionally relevant partners. A popular proposal places PsbS in the cleft between LHCII trimer M and the PSII core (Fig. 2; Su et al. 2017), but this hypothesis is still awaiting experimental validation.

### Is PsbS essential for NPQ?

In vivo, under physiological conditions, the answer is undoubtedly yes. In the absence of PsbS, only minimal fluorescence quenching is observed (Li et al. 2000), corresponding to an NPQ value of 0.2 to 0.3 in *A. thaliana*. However, it was demonstrated that artificially increasing the ΔpH across the thylakoid membrane to nonphysiological values could restore NPQ in the absence of PsbS (Johnson and Ruban 2011). This result supports the conclusion that PsbS is not the quencher itself but rather acts as an allosteric effector that controls the conformational switch of LHCs, permitting it to occur under physiological pH conditions.

### How many PsbS proteins are present per PSII?

The exact stoichiometry of PsbS relative to PSII remains unresolved. It was initially suggested that PsbS is present in a 1:1 ratio with the PSII core subunit PsbO (Funk et al. 1995), but recent data reported approximately 1 PsbS per 2 PSII supercomplexes (McKenzie et al. 2020) or even 1 every 4 PSII (Hu 2024). Despite variability in the data, a consistent observation across studies is that the amount of PsbS is significantly lower than that of LHCII.

Another critical point is that the 2 Glu residues whose protonation is essential for PsbS activation (Li et al. 2004) have pKa values of 5.2 and 6.1 (Liguori et al. 2019). Since the thylakoid lumen typically does not reach pH values <5.5 (Kramer et al. 1999 ; Tikhonov et al. 2008) even under high-light conditions, <30% of the PsbS proteins in the membrane are expected to be fully protonated and thus active. This further lowers the effective ratio of active PsbS to PSII.

An implication of the low abundance of PsbS is that, if it is directly involved in forming the quencher (e.g. by association with LHCII), the resulting quenching must be highly efficient.

## Role of Zx in NPQ

The involvement of Zx in NPQ was first reported by Demmig and coworkers, who showed that the conversion of Vx into Zx linearly correlates with the level of NPQ (Demmig et al. 1987; Demmig-Adams et al. 1989; Gilmore and Yamamoto 1993). This reversible conversion via the intermediate antheraxanthin constitutes the xanthophyll or VAZ cycle (Yamamoto et al. 1962) and is triggered by the low lumenal pH, which activates the enzyme VDE, the activity of which rises sharply going from pH 6.3 to 5.8 (Pfundel and Dilley 1993). When ΔpH drops, Zx is slowly converted back into Vx by the enzyme Zx-epoxidase, present in the stroma (Marin et al. 1996). The synthesis of Zx occurs largely on a time scale of 5 to 10 min (Jahns and Holzwarth 2012; Kuster et al. 2023; Ramakers et al. 2025b), meaning that Zx does not dominate the initial fast NPQ induction but is largely responsible for a slower rise component. During a subsequent period of darkness, NPQ relaxes to a large extent within 1 to 2 min, despite the much slower epoxidation of Zx (Hartel et al. 1996), indicating that the presence of Zx alone is not sufficient to maintain quenching. When the light is switched on again, qE is reactivated more rapidly and at lower ΔpH, which was historically attributed to residual Zx from the previous light period (see e.g. Rees et al. 1989; Ruban and Horton 1999; Nilkens et al. 2010). However, it was recently demonstrated that the rapid reactivation is not due to Zx but to PsbS (Ramakers et al. 2025a). The rise of NPQ is equally fast for the *npq1* mutant (which cannot de-epoxidize Vx and no Zx is formed) as it is for WT and the *npq2* mutant (where Zx is constitutively present), although the amplitude of NPQ is significantly smaller. Therefore, Zx might be considered to amplify the amount of NPQ induced by protonated PsbS. In addition, Zx is responsible for a slowly induced quenching component that is missing in the absence of PsbS (Li et al. 2000). Thus, Zx contributes to the amplitude of rapid qE and a distinct slower phase (often referred to as qZ), in both cases requiring PsbS, and it is clear that PsbS and Zx need each other to create substantial amounts of NPQ.

### Is Zx essential for NPQ?

As stated previously, Zx is strictly speaking maybe not essential because some NPQ occurs in the npq1 mutant of *A. thaliana* in which Zx is not produced. However, the amount of NPQ in that mutant is substantially smaller (qE = ∼0.5; Niyogi et al. 1998) than in WT plants, so Zx is certainly important for NPQ and amplifies the effect of protonated PsbS to a large extent. It also provides a sort of longer-term memory effect for repeated exposure to high light (Kromdijk et al. 2022). Whereas PsbS can take care of relatively fast fluctuations in light intensities, Zx is available for long-term fluctuations, for which PsbS is also important.

### Where is Zx located?

The location and binding of Zx within the photosynthetic apparatus have long been debated, largely because early hypotheses proposed that Zx itself acted as the quencher and then its localization would lead to the identification of the quenching complex. Initial studies suggested that Zx replaces Vx in the internal binding sites of the minor antenna complexes CP24, CP26, and CP29 (Morosinotto et al. 2002; Dall’Osto et al. 2005) but also at the external xanthophyll binding site of LHCII (Ruban and Horton 1999). Indeed, Zx can bind to all antenna complexes when present during folding in vitro and in vivo (Jahns et al. 2001; Morosinotto et al. 2002). However, under physiological conditions, it was found to be mainly associated with LHCII in its external binding site (Xu et al. 2015). Despite this association, Zx does not induce quenching in isolated LHCII (Xu et al. 2015), casting doubt on the idea that it acts as a direct quencher within the antenna, in the absence of PsbS. Instead, a large pool of Zx appears to be in the lipid phase of the thylakoid membrane, where it likely serves as an antioxidant (Havaux et al. 2007) and possibly performs its role as an allosteric activator (Perez-Bueno et al. 2008). The situation might be different in mutants in which Lut is not present, such as *lut2* (Pogson et al. 1998), as Zx might replace Lut in the internal binding sites of the LHCs. In that case, PsbS might induce quenching in LHCs via the internal Zx molecules. Induction of NPQ in those mutants shows indeed a substantial amount of quenching (Lee et al. 2024). Apart from quenching in the Lut positions, Zx would still fulfil its role as an amplifier. It was concluded that Zx is a better quencher than Lut because NPQ decreases more in the absence of Zx than of Lut (Lee et al. 2024). In our view, a likely explanation for this difference in quenching capacity is the fact that Zx can partly take over the role of internal quencher from Lut in the absence of the latter, whereas Lut cannot take over the role of quencher amplifier from Zx.

One additional possibility to consider is that Zx can be located at the interface between proteins, creating new quenching sites. However, at present there is no experimental evidence for this hypothesis.

In conclusion, the action mechanism of PsbS and Zx in NPQ remains unsolved. Yet, their cooperative function is evident in fast and slow phases of NPQ. The observation that PsbS acts more rapidly after the first light cycle strongly suggests that it largely remains positioned within the quenching site, whereas Zx assists in enhancing its effect.

To understand how PsbS and Zx translate a pH signal into effective quenching, it is essential to consider the membrane environment in which NPQ takes place.

## Big picture

### How can the low lumenal pH lead to quenching in the membrane?

As discussed earlier, the low pH alone at physiological pH values (>5.5) is not sufficient to induce quenching in monomeric or trimeric LHCII, neither in detergent solution (Liguori et al. 2013) nor in nanodiscs (Ruan et al. 2023). Instead, quenching can easily be induced in vitro by clustering LHCII in liposomes (Natali et al. 2016; Tutkus et al. 2021) or promoting aggregation in low-detergent conditions (Horton et al. 1991; Barzda et al. 2001), which can be enhanced when the pH is close to LHCII's isoelectric point (4.5 to 5). In vivo, under physiological conditions, full activation of NPQ requires PsbS and Zx, indicating that they link the low lumenal pH to the quenching. It has been proposed that PsbS and Zx influence LHCII aggregation and/or its affinity for protons (Horton et al. 2008; Horton 2012). PsbS has also been implicated in regulating the overall organization of PSII (Kiss et al. 2008; Kereiche et al. 2010; Goral et al. 2012; Dong et al. 2015), but no study has directly demonstrated a reorganization of membrane protein architecture in the short time scale of NPQ induction upon PsbS protonation or lowering of the lumenal pH.

### Are membrane reorganization and LHCII aggregation responsible for NPQ in vivo?

While LHCII aggregation is a well-established trigger of quenching in vitro (Ruban and Horton 1992; van Oort et al. 2007a), proving that aggregation occurs in vivo in physiological conditions and is directly responsible for NPQ in plants is considerably more challenging. In the following sections, we critically examine the evidence for and against the idea that LHCII aggregation drives NPQ under physiological conditions.

### What is the putative spectroscopic signature for aggregation?

The hypothesis that LHCII aggregation is responsible for qE was originally proposed by Horton and coworkers based on the similarities between the 77-K fluorescence spectra of leaves and LHCII aggregates. Induction of NPQ in leaves at room temperature, followed by fluorescence measurements at 77 K, showed a relative increase of a fluorescence peak around 700 nm when compared with fluorescence at 680 nm (Ruban et al. 1993). Because LHCII aggregation also leads to red-shifted fluorescence (Ruban and Horton 1992), this shift was taken as an indication for aggregation-induced NPQ (see Johnson and Ruban 2009). However, the red-shifted feature does not appear immediately. It becomes noticeable only after several minutes of illumination, well after the main phase of qE is already established (Ruban et al. 1993). Moreover, 77-K fluorescence spectra from leaves are difficult to interpret, due to overlapping signals, self-absorption, and a large increase of PSI fluorescence, especially >700 nm. Proper normalization of these steady-state spectra and the separation of PSI and PSII contributions are technically challenging and somewhat arbitrary. At room temperature, red-shifted emission (now around 725 nm instead of 700 nm) was reported by Holzwarth et al. (Miloslavina et al. 2008), using picosecond fluorescence spectroscopy on quenched *A. thaliana* leaves. Yet, these spectra were substantially influenced by self-absorption. A later study that minimized self-absorption (Farooq et al. 2018) confirmed the presence of a far-red fluorescence band around 725 nm, but it was present only when the RCs were closed. When the RCs were open, the far-red band disappeared almost instantaneously and reappeared upon RC closure within a millisecond (van Amerongen and Chmeliov 2019). This dynamic behavior is inconsistent with a static phenomenon such as LHCII aggregation and suggests that the 725-nm band does not reflect a quenched, aggregated state of LHCII. Additional support for aggregation-based quenching came from resonance Raman spectroscopy: changes in the Nx spectral region in LHCII aggregates was found to match spectra from quenched LHCII crystals and WT Arabidopsis leaves in which qE had been induced (Pascal et al. 2005; Ruban et al. 2007). However, a more recent study showed that the same spectral signature also appears in LHCII preparations that are not quenched (Li et al. 2021).

Altogether, these observations suggest that while LHCII aggregation is strongly associated with quenching in vitro, its spectroscopic signatures are not reliable indicators of qE in vivo.

### Is there structural or biochemical evidence for reorganization or aggregation of photosynthetic complexes during NPQ?

The main argument in favor of membrane reorganization during NPQ comes from electron microscopy studies that reported differences in the density of PSII complexes in the membrane between light- and dark-adapted plants and changes dependent on the presence of Zx (Betterle et al. 2009; Johnson et al. 2011b). These observations were interpreted as disconnection of LHCII from PSII, followed by LHCII aggregation, leading to quenching. Partitioning of LHCII in the membrane was supported by freeze fracture data, which identified LHCII-only domains in quenching conditions (Johnson et al. 2011b). Building on this, it was proposed (Johnson et al. 2011a) that protonation of PsbS induces lipid rearrangement around LHCII, leading to membrane thinning and hydrophobic mismatch that possibly separates LHCII from its environment and drives it into photoprotective nanodomains (see Ruban and Wilson 2021; Navakoudis et al. 2023; Wilson et al. 2024). Reduced mobility of some complexes detected by FRAP experiments (Goral et al. 2010) was interpreted as supporting aggregation of detached LHCII during NPQ. These interpretations were supported by biochemical (Betterle et al. 2009) and spectroscopic (Holzwarth et al. 2009) studies suggesting a disconnection of LHCII from the PSII core. However, an important factor that does not seem to match is the time scale of events: for instance, Betterle et al. (2009) observed PSII supercomplex disassembly in membranes prepared in the dark, a condition in which qE is already relaxed. Similarly, Johnson et al. (2011b) still observed clustering after 5 min of dark adaptation in the presence of Zx, a condition in which there is almost no quenching left. Yet, there is no clear evidence supporting the reorganization of the membrane during the fast qE induction (<1 min), and biochemical data did not detect disassembly of PSII supercomplexes during quenching (Bielczynski et al. 2022).

Altogether, these results suggest that large-scale reorganization of the membrane is not required to initiate quenching. However, structural rearrangements may still play a role in facilitating or stabilizing NPQ once it has formed.

In conclusion, while the idea is widely spread that PsbS and Zx favor reorganization of the complexes in the membrane, creating quenching, essential proof to support the scenario that the quenching is caused by aggregation of LHCII is still missing.

To better evaluate the possible grand NPQ scenarios, it is useful to have at least a semiquantitative understanding of the number of quenching centers needed to match observed quenching levels. This topic is addressed in the next section.

### How many quenchers are needed to quantitatively explain the amount of NPQ?

The level of NPQ can vary a lot, depending on various factors, but to get a feeling for the required number of quenchers, we take a value of NPQ = 2, which is typical for *A. thaliana* grown at a light intensity of ∼100 μmol m^−2^·s^−1^. Because NPQ = *k*_NPQ_ × τ*_m_* (see Appendix) and the excited-state lifetime τ_m_ for closed PSII RCs is ∼2 ns (Belgio et al. 2012), the average rate of NPQ, *k*_NPQ_, is ∼1.0 ns^−1^. This rate can conveniently be compared with the rate of photochemical quenching by open PSII RCs. The average excited-state lifetime of PSII for Arabidopsis grown in similar light conditions was reported to be ∼225 ps with on average ∼2.4 LHCII trimers present per PSII RC (Wientjes et al. 2013b) and thus 111 Chls *a*. This corresponds to a rate of photochemical quenching of *k*_PQ_ = (0.225 ns)^−1^ – (2 ns)^−1^ = ∼4.0 ns^−1^, 4 times as large as the value of *k*_NPQ_ = ∼1.0 ns^−1^. We now make a distinction between the case in which the quencher is either fast or slow, and as examples we use the two types of quenched conformations that LHCII trimers can adopt under high pressure (van Oort et al. 2007b) (see section: How many quenchers are needed to quantitatively explain the amount of NPQ?): one with a fluorescence lifetime of 25 ps (fast quenching) and one with the ∼500-ps lifetime (slow quenching).

In case of a fast quencher, *k*_NPQ_ and *k*_PQ_ are to a large extent determined by the migration time that is needed for an excitation to reach the photochemical or nonphotochemical quencher (Chmeliov et al. 2014). This migration time scales linearly with the number of excitation hops between pigments (on a regular 2D lattice; i.e. *d* = 2.0) and thus with the number of pigments per trap (Chmeliov et al. 2014). Note that PSII was found to effectively act as a 2D lattice with *d* = 1.9 (Farooq et al. 2018). This implies that 1 nonphotochemical quencher per 4 PSII RCs would be sufficient to obtain a value of NPQ = 2, provided that excitations can move freely between photosystems. Note that a doubling of the number of quenchers leads to a doubling of NPQ (NPQ = 4), while still only 1 quencher per 2 RCs is required. Only for very high values of NPQ, for instance NPQ = 8, approximately 1 nonphotochemical quencher per RC would be required. However, in many cases, a substoichiometric number of nonphotochemical quenchers, as compared with the number of RCs, is sufficient to obtain realistic numbers for the NPQ parameter.

In case of a slow quencher (∼500 ps in our example), NPQ becomes far less efficient, and in that case around ∼2.7 quenchers per RC would be needed to reach a value of NPQ = 2 (see Appendix)—that is, >10 times as many quenchers to reach the same amount of quenching.

### What are the implications of the number of quenchers per RC for the quenching scenarios?

As discussed earlier, protonated PsbS has to move to a place where it can trigger NPQ, presumably in one of the LHCs. Given that PsbS is substoichiometric relative to PSII RCs, it must act efficiently, activating a highly effective quencher. This could, for instance, be the 25-ps conformation of trimeric LHCII (van Oort et al. 2007b) or the fast-quenching conformation of CP29, where a twisted Lut functions as a quencher (Mascoli et al. 2019). The substoichiometric nature of PsbS also implies that Zx cannot enhance quenching by simply increasing the quenching speed at the same site, since this is already very fast and the quenching is mainly limited by excitation diffusion through the PSII antenna system to the quencher (Chmeliov et al. 2014). This would imply that Zx can increase the overall rate of quenching only by (i) creating extra quenching centers or (ii) creating better connectivity between the quenched LHC and the rest of PSII, acting as “molecular glue.” This would make the PsbS-induced quencher accessible to a larger number of LHCs, thereby increasing the “capture area” of the quencher. Given that PsbS is substoichiometric with respect to the number of supercomplexes, this glueing effect of Zx could be a highly efficient amplification mechanism. It has indeed been shown experimentally that Zx can mediate interactions between LHCs (Gruszecki et al. 2006; Zhou et al. 2020). It is interesting to note that Belgio et al. (2014) also concluded that the effective antenna size of PSII increased upon induction of NPQ. As those authors pointed out, this leads to economic photoprotection, meaning that despite the quenching of excitations due to NPQ, the number of excitations trapped in open RCs can even increase. The main difference between their model and our model is the number of quenchers per photosystem. They conclude that there are many slow quenchers, whereas we conclude/propose that there is only a substoichiometric number of very fast quenchers that are induced by protonated PsbS, whereas the main role of Zx is to increase the functional antenna size, thereby creating economic photoprotection in an alternative way.

## Summary and our proposal for a working model for qE in plants

**Figure 6.**
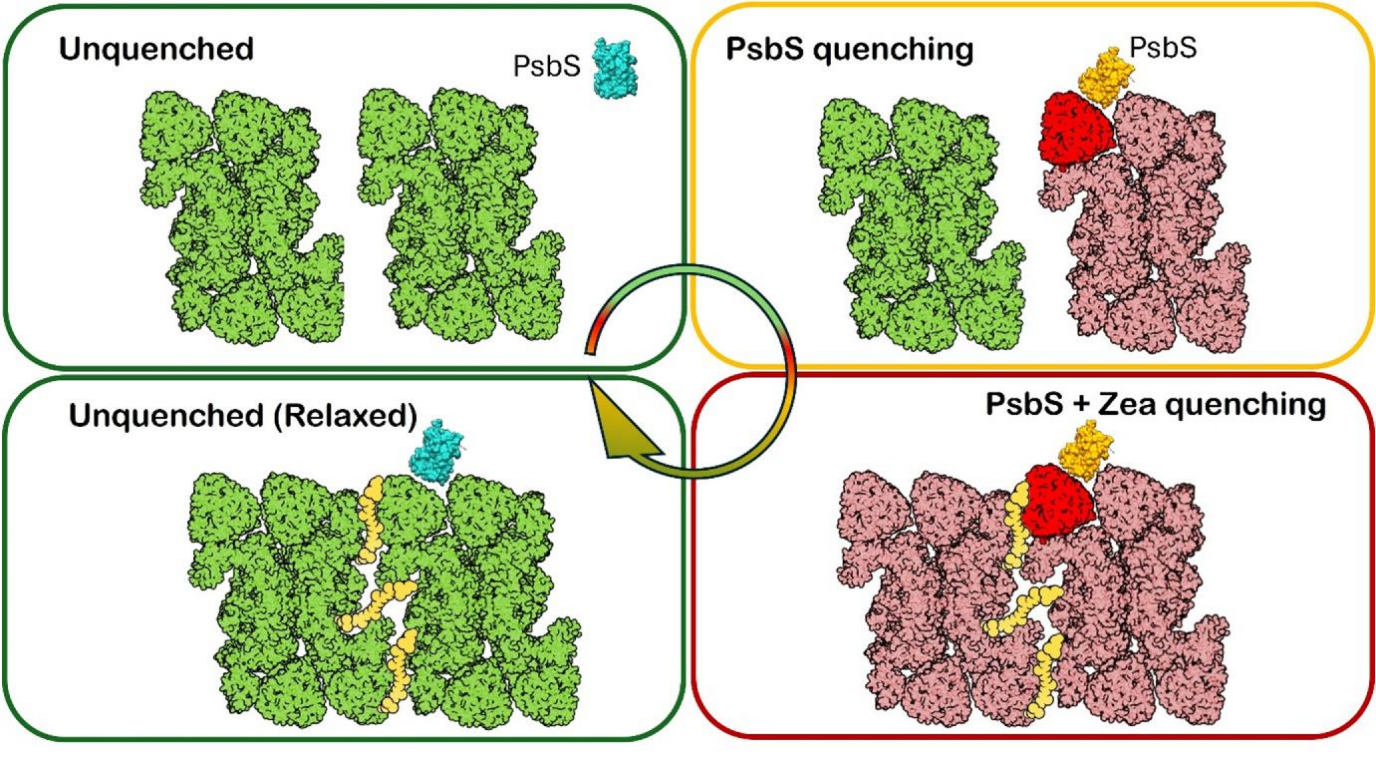
Cartoon of the proposed mechanism of the first minutes of NPQ and its relaxation. In the upper left figure, the 2 PSII supercomplexes are not quenched (green); PsbS is not protonated (blue); and it is not specifically bound somewhere in one of the supercomplexes. The supercomplexes are badly connected to each other in this model, as in the puddle model. In the next cartoon (top right), NPQ has just been induced, and PsbS is protonated (yellow) and binds to an LHC—in this case, LHCII—and turns it into a quencher (red) that quenches the rest of the supercomplex (light red). The other supercomplex is not connected and does not get quenched. As a next step, Zx (yellow) is being formed and connects the 2 supercomplexes, also leading to quenching of the other supercomplex (bottom right). Immediately after the light intensity is decreased (bottom left), PsbS is deprotonated; it does not induce quenching anymore in LHCII; and the quenching of both supercomplexes disappears, although PsbS and Zx are still in place. It is important to realize that in this cartoon Zx increases the quenched region by connecting an unquenched supercomplex to a quenched one, but this should not be taken too literally. For an increase of the quenched region, other scenarios might be envisaged. Note that Zx is not at scale, but it is a cartoon representation to show how we speculate that it might work.

On the basis of this discussion, we propose the following working model for qE in plants (see also Fig. 6): A drop in lumenal pH places LHCII and/or a minor complex in a poised but nonquenched state. Upon protonation, PsbS binds to one of these complexes and induces a conformational change, thereby turning it into a quencher. At the molecular level, the quenching is driven by incoherent EET from a red-shifted Chl *a* cluster to a so-called S* state of Lut 1, which is the first excited state in a twisted conformation that can dissipate energy nonradiatively. Because PsbS is substoichiometric relative to PSII and its antenna, it must selectively induce fast and efficient quenchers. These might include, for example, the 25-ps quenched conformation of trimeric LHCII (van Oort et al. 2007b) or the quenched state of CP29 (Mascoli et al. 2019). Due to the migration-limited nature of excitation energy flow in the PSII antenna network, additional quenching induced by Zx cannot simply speed up the quenching at these sites. Instead, we propose that Zx amplifies NPQ by improving the energetic connectivity between PSII antenna complexes—for instance, by promoting lateral energy transfer between adjacent PSII supercomplexes. This allows excitations to be more efficiently transferred toward quenched antennae, thus increasing the overall NPQ amplitude.

Quantitatively, our model predicts that 1 PsbS per 4 RCs is sufficient to achieve an NPQ value of ∼2, assuming that each PSII RC is connected to 2 or 3 LHCII trimers. Because NPQ scales roughly linearly with the number of quenchers, doubling the amount of PsbS (e.g. 2 per 4 RCs) would yield an NPQ value of ∼4. This is consistent with experimental observations in Arabidopsis mutants with PsbS overexpression, which exhibit enhanced quenching (Li et al. 2002a; Logan et al. 2008; Glowacka et al. 2018) when corrected for PSII activity and the presence of PSI.

Importantly, once PsbS and Zx are positioned correctly in the membrane, subsequent NPQ activation after a dark period is rapid, reflecting the persistence of a structurally primed quenching state (Rees et al. 1989; Townsend et al. 2018). While the core mechanism is likely dominated by fast, localized conformational switching, additional, slower reorganization events in the membrane may contribute to sustained quenching phases or to long-term photoprotective states.

Although our model can explain many available data, we recognize that it remains a working model that needs to be verified and improved but can also be falsified. However, its level of detail allows for specific testable predictions, and we hope that it will stimulate experimental work aimed at testing one or more of the proposed aspects.

## Data Availability

NA.

## Appendix

The following materials are available in the online version of this article.

### Useful relations between fluorescence parameters and rate constants

The basic fluorescence parameters often measured for dark-adapted plants are *F*_0_ (fluorescence with supposedly all PSII RCs open) and *F_m_* (with all RCs closed after a saturating light flash). These parameters relate to underlying rate constants as (also called the Butler model after Butler 1978):

$$F_{0}=C\times k_{F}/(k_{F}+k_{\mathrm{IC}}+k_{\mathrm{ISC}}+k_{\mathrm{phot}})$$

and

$$F_{m}=C\times k_{F}/(k_{F}+k_{\mathrm{IC}}+k_{\mathrm{ISC}}),$$

where the rate constants are defined as follows: *k_F_* = (average) rate of fluorescence or radiative rate of the Chls in PSII, *k*_IC_ = rate of decay via heat production (internal conversion in the absence of NPQ), *k*_ISC_ = rate of intersystem crossing (or triplet formation), and *k*_phot_ = rate of photochemistry.

*C* is a constant depending on the specifics of the setup, and it can be left out because in all relevant cases a ratio of fluorescence intensities is taken, thereby canceling out *C*.

A parameter commonly determined is *F_v_*/*F_m_* = (*F_m_* – *F_o_*)/*F_m_*. It is equal to *k*_phot_/(*k_F_* + *k*_IC_ + *k*_ISC_ + *k*_phot_)—that is, the fraction of excitations used for photosynthesis in PSII. For healthy plants, a typical value of *F_v_*/*F_m_* is 0.83. This value of 0.83 is also used when applying the method of Kramer and coworkers (Tietz et al. 2017) to estimate NPQ in the field—namely, NPQ_(T)_. It should be kept in mind that the fluorescence that is measured also contains a contribution from PSI, and if this contribution is subtracted from the measured fluorescence intensities, the value of *F_v_*/*F_m_* increases by several percentage points and the typical value of 0.83 increases to approximately 0.87–0.88.

After induction of NPQ, the decay rate of NPQ, *k*_NPQ_, also has to be included in the previous expressions, and one obtains $F_{0}^{\prime }=k_{F}/(k_{F}+k_{\mathrm{IC}}+k_{\mathrm{ISC}}+k_{\mathrm{phot}}+k_{\mathrm{NPQ}})$ and $F_{m}^{\prime }=k_{F}/(k_{F}+k_{\mathrm{IC}}+k_{\mathrm{ISC}}+k_{\mathrm{NPQ}})$, where $F_{0}^{\prime }\;\mathrm{and}\;F_{m}^{\prime }$ are measured like *F*_0_ and *F_m_* but now in the presence of actinic light. Note that *C* has already been omitted. Here *k*_NPQ_ is the rate of excited-state decay due to NPQ.

Instead of using fluorescence intensity methods, time-resolved fluorescence measurements can be used to determine various parameters and in particular rate constants. The average excited-state lifetime τ_0_ of PSII in *F*_0_ conditions is equal to $1/(k_{F}+k_{\mathrm{IC}}+k_{\mathrm{ISC}}+k_{\mathrm{phot}})$, and it is typically several hundreds of picoseconds (Croce and van Amerongen 2020). The average excited-state lifetime τ*_m_* of PSII in *F_m_* conditions is equal to $1/(k_{F}+k_{\mathrm{IC}}+k_{\mathrm{ISC}})$ and is around 2 ns (Belgio et al. 2012). In the presence of NPQ, these lifetimes change and become $\tau_{0}^{\prime }=1/(k_{F}+k_{\mathrm{IC}}+k_{\mathrm{ISC}}+k_{\mathrm{phot}}+k_{\mathrm{NPQ}})$ and $\tau_{m}^{\prime }=1/(k_{F}+k_{\mathrm{IC}}+k_{\mathrm{ISC}}+k_{\mathrm{NPQ}})$. Another helpful equation is

$$\begin{array}{rl} \mathrm{NPQ} & =(F_{m}-{F^{\prime }}_{m})/F_{m}^{\prime }=k_{\mathrm{NPQ}}/(k_{F}+k_{\mathrm{IC}}+k_{\mathrm{ISC}})\\ & =k_{\mathrm{NPQ}}/(k_{F}+k_{d})=k_{\mathrm{NPQ}}\times \tau_{m}. \end{array}$$

As mentioned, all these derivations hold for PSII, excluding fluorescence contributions from PSI. However, PSI contributes to the fluorescence, especially in the far-red region (>700 nm), which is exactly the detection range used in PAM measurements. Although the contribution of PSI at physiological temperatures is considered to be minor due to the very short excited-state lifetime of the complex in the reduced and oxidized states, it is influenced by the PSI/PSII ratio and can thus be more relevant in some plants/mutants than others. For quantitative analysis, it is thus necessary to correct for the PSI contribution. This can be done, for example, by using a streak camera setup to obtain time- and spectrally resolved fluorescence data because PSI and PSII have distinct spectral and temporal (ps–ns time scale) features (see e.g. Farooq et al. 2018). By analyzing the time- and spectrally resolved fluorescence data, one can get a good estimate of the fluorescence spectra of PSI and PSII and the corresponding average fluorescence lifetimes. The overall steady-state fluorescence spectrum can then be calculated by taking the product of the PSI spectrum and its average lifetime (PSI contribution) and adding it to the product of the PSII spectrum and its average lifetime (PSII contribution). The contribution of PSII depends on the state of its RC—that is, open (*F*_0_) or closed (*F_m_*)—and the corresponding average lifetimes are typically 150 to 400 ps depending on antenna size (van Oort et al. 2010; Caffarri et al. 2011; Wientjes et al. 2013b) and 2 ns (Belgio et al. 2012). The average lifetime of PSI, however, is virtually independent of the state of the RC, and it is typically around 50 to 100 ps for plants, depending on its antenna size (Croce and van Amerongen 2020). A crude estimate of the PSI contribution to *F*_0_ is around 30%. In an absolute sense, the contribution of PSI to *F_m_* remains the same, but percentage-wise, it goes down to only a few percentage points. Of course, the relative contributions of PSI and PSII depend on excitation and detection wavelengths.

### How many slow quenchers are needed per PSII RC to obtain a value of NPQ = 2?

As an example of a slow quencher, we take trimeric LHCII with a lifetime of 500 ps specifically, one of the two quenched conformations found at high hydrostatic pressure (van Oort et al. 2007b). Because the concomitant unquenched lifetime is 3.5 ns, the rate of quenching for such a trimer is (1/0.5 ns) – (1/3.5 ns) = 1.7 ns^−1^. Since this quenching is slow, it is not migration limited as in the case of the fast 25-ps conformation, but it is nearly trap limited. Because a trimer contains 24 Chls *a* whereas PSII on average contains 111 Chls *a* in our example, the rate of quenching caused by one such trimer per PSII RC becomes (24/111) × 1.7 ns^−1^ = 0.37 ns^−1^. As pointed out in the main text, we would need a rate of *k*_NPQ_ = 1 ns^−1^ to obtain a value of NPQ = 2. Therefore, we would need approximately 1/0.37 = 2.7 quenched trimers per PSII RC to reach an overall value of NPQ = 2.
